# Alginate/κ-Carrageenan-Based Edible Films Incorporated with Clove Essential Oil: Physico-Chemical Characterization and Antioxidant-Antimicrobial Activity

**DOI:** 10.3390/polym13030354

**Published:** 2021-01-22

**Authors:** Aji Prasetyaningrum, Dani P. Utomo, Al Farrel A. Raemas, Tutuk D. Kusworo, Bakti Jos, Mohammad Djaeni

**Affiliations:** 1 Department of Chemical Engineering, Faculty of Engineering, Diponegoro University, Jl. Prof. Soedarto S.H., Tembalang, Semarang 50275, Indonesia; danipujiutomo@che.undip.ac.id (D.P.U.); farrelraemas@students.undip.ac.id (A.F.A.R.); tdkusworo@che.undip.ac.id (T.D.K.); baktijos@che.undip.ac.id (B.J.); moh.djaeni@live.undip.ac.id (M.D.); 2 Membrane Research Center (MeR-C), Diponegoro University, Jl. Prof. Soedarto S.H., Tembalang, Semarang 50275, Indonesia

**Keywords:** alginate, antimicrobial activity, antioxidant, carrageenan, clove essential oil, edible films, polysaccharide

## Abstract

This study aimed to enhance the properties of CaCl_2_ crosslinked sodium alginate/k-carrageenan (SA/KC) incorporated with clove essential oil (CEO). An evaluation of the modification effects on physicochemical, morphological, antioxidant, and antibacterial properties was performed. The properties were observed at various SA/KC ratios (10/0 to 1.5/1), CEO (1.5% to 3%), and CaCl_2_ (0% to 2%). The surface morphology was improved by addition of KC and CaCl_2_. The Fourier transform infrared (FTIR) result showed insignificant alteration of film chemical structure. The X-ray diffraction (XRD) result confirmed the increased crystallinity index of the film by CaCl_2_ addition. On physicochemical properties, a higher proportion of SA/KC showed the declined tensile strength, meanwhile both elongation at break and water solubility were increased. The incorporated CEO film reduced both tensile strength and water solubility; however, the elongation at break was significantly increased. The presence of Ca^2+^ ions remarkably increased the tensile strength despite decreased water solubility. Overall, the addition of KC and CaCl_2_ helped in repairing the mechanical properties and flexibility. CEO incorporation showed the effectiveness of profiling the antioxidant and antimicrobial activity indicated by high 2,2-diphenyl-1-picrylhydrazyl (DPPH) scavenging activity up to 90.32% and inhibition zone of *E. coli* growth up to 113.14 mm^2^.

## 1. Introduction

Marine resources such as macroalgae have been an essential source of polysaccharide-based biomaterials for many years. The development of bioactive films from seaweed has gained considerable attention due to their renewability, low toxicity, biocompatibility, biodegradability, and potential function as food packaging [1,2]. These packaging films could increase food preservation to improve the stability of food and protect the product from deterioration due to environmental conditions [3,4]. Biopolymer-based film products can be developed to produce environment-friendly materials. The utilization of renewable materials to produce edible films can reduce waste disposal issues. The development of edible films containing natural polysaccharides and essential oil becomes an interesting study for maintaining food quality and providing protection against the oxidation process, also minimizing the pathogen contamination [3,5,6]. There are three major types of polysaccharide that have been widely utilized from marine sources for edible film production: alginates, carrageenan, and agar.

**Figure 1:**
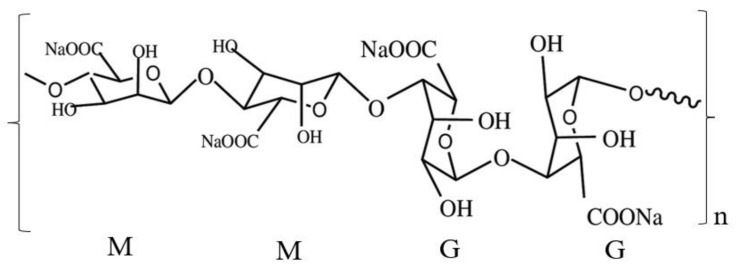
Structural characteristics of alginates: β-D-mannuronic acid (M) and α-L-guluronic acid (G) residues.

Alginate is a natural polysaccharide extracted from brown algae (*Sargassum* sp.), which is interesting to be developed as a bio-edible film due to its versatility, nontoxicity, biocompatibility, gel-forming capability, and biodegradable [1,7]. The chemical structure of alginate consists of *β*-(1,4)-linked *D*-mannuronic acid units (M) and *L*-guluronic acid units (G) [1,7,8]. Alginate is a natural polysaccharide containing carboxyl groups in its constituent residues and various capabilities for application as functional materials [9]. Alginate-based bio-composites have been used widely in a few industries, such as food packaging, biomedical, tissue-based materials, and pharmaceutical fields. However, alginate has several drawbacks, such as low water resistance due to hydrophobicity behavior [9]. The chemical structure of alginate is presented in Figure 1 [10].

Many studies have been reported about the combination of alginates and other polysaccharides to improve the edible film [1,2,5]. The development of alginate-based edible films incorporated with another natural polysaccharide into the alginate matrix could increase the physical and mechanical properties. *κ*-carrageenan becomes a promising material to be combined with alginate to achieve better film characteristics. Carrageenan is a generic name for a natural polysaccharide extracted from the primary cell wall of red algae (*Rhodophyta*). Carrageenan contains water-soluble hydrocolloids of a linear chain of sulfated galactans. It has been recognized from the formula and chain position of a *β*-(1,3) sulfated D-galactose and *α*-(1,4)-3,6-anhydrous-D-galactose residue [3,4]. Kim et al. [11] reported that carrageenan could make transparent films with excellent mechanical and physical properties. However, natural polysaccharides generally have low water vapor barrier properties due to their hydrophilicity. Furthermore, the addition of lipids, such as clove essential oil, is expected to improve physical and mechanical properties [3].

The application of essential oil into edible films could increase the physical and mechanical properties. Spice essential oils extracted from plants such as clove, curcumin, lemongrass, ginger, thyme, cinnamon, turmeric, garlic, etc. have been studied for their antimicrobial, antifungal, and antiviral activities [12]. Recent studies have confirmed that more than 67 spice essential oil extracts show in vitro antibacterial activity against pathogenic bacteria [13]. Clove essential oil (CEO) could be a good potential for natural resources and recover properties by expanding flavouring substances with antimicrobial, insecticidal, antifungal, and antioxidant activities [14]. Clove essential oil is extracted from dried flower buds of *Syzygium aromaticum* containing various bioactive compounds [15]. Eugenol (4-allyl-2-methoxy phenol) is the main bioactive ingredient in essential oil. Mulla et al. [15] have infused the CEO in the linear low-density polyethylene to improve ultraviolet (UV)-barrier properties and antimicrobial activity against pathogenic bacteria. Ahmed et al. [14] have reported that the essential oil blend in the polylactide-based polymer significantly improved immunity against selected pathogens, such as *S. Typhimurium* and *L. monocytogenes*.

Alginate-based edible films can react with di- and trivalent cations, specifically calcium ions. The use of alginate-based edible films crosslinked with Ca^2+^ ions has been reported in improving the overall capacity and functionality of the edible film. Li and co-workers [16] have revealed that the crosslinking of sodium alginate using Ca^2+^ ions in the glucomannan matrix significantly improved the thermal stability, surface hydrophobicity, and tensile strength of the films. The improvement of alginate-based film properties using Ca^2+^ crosslinking was also reported by Giz and co-workers [17]. The Ca crosslinking has shown remarkable enhancement in terms of thermal stability, anti-swelling degree, and vapor permeability. Lee et al. [18] studied the gelation process of alginate film via spraying Ca^2+^ ion droplets, and they found that the gelation degree was dependent on both alginate and Ca concentrations. However, to the best of the authors’ knowledge, no previous studies have demonstrated the effect of different ratios of sodium alginate/κ-carrageenan (SA/KC)-based films with the addition of CEO and Ca^2+^ crosslinking interaction on different types of edible film structure.

Therefore, this work mainly aimed to simultaneously increase the strength and properties of SA/KC films with CaCl_2_ as crosslinker and CEO as antimicrobial agent. Tensile strength and water solubility were evaluated in this study to assess the mechanical and physical properties. The morphology, chemical structure, and crystallinity behaviours of the composite films were characterized. Furthermore, the unique properties of composite films, such as antimicrobial and antioxidant activity, were evaluated as a potential food packaging material.

## 2. Materials and Methods

### 2.1. Materials

Sodium alginate (SA) (CAS No. 9005-38-3, #, purity ≥ 98%) was obtained from Sigma-Aldrich, Darmstadt, Germany. Semi-refined κ-carrageenan was supplied by CV. Karaindo, Semarang, Indonesia. Clove essential oil (CEO) (Eugenol content 81.29 wt.-%) extracted from clove bud (*Syzygium aromaticum*) was purchased from Perkebunan Nusantara, Ltd., Purwokerto, Indonesia. Furthermore, food-grade anhydrous CaCl_2_ pellets (purity 94–97%) were obtained from OxyChem, Dallas, TX, USA.

### 2.2. Edible Film Preparation

Film-forming solutions of SA/KC powder with various ratios (10/0; 9/1; 8/2; 7/3; 6/4) were dissolved in 200 mL of distilled water, stirred at 250 rpm for 1 hour, and temperature adjusted at 50 °C. When the solution reached homogeneously, an appropriate amount of CEO (varied at 1.5, 2.0, 2.5, and 3.0% *v*/*v*) was added slowly into the film solution, and the stirring continued for another hour. Subsequently, the film solution was cooled down to 30 °C to remove air bubbles and dissolved air [7]. Then the solution was cast onto the center of a clean glass plate and dried at room temperature for 30 h [4]. The dried films were removed from the casting surfaces and stored in desiccators at 25 °C and 87% relative humidity. All films were peeled from the casting plates and held under the same conditions for a further 12 h prior to characterization procedures [9]. Furthermore, the formed film was crosslinked with CaCl_2_ solution at various concentrations (0%, 1%, and 2% *w*/*v*). A pristine SA film was also prepared as a control during characterization and property evaluation.

### 2.3. Film Characterizations

#### 2.3.1. Morphology Observation Using Scanning Electron Microscopy (SEM)

The microscopic morphology of composite films was observed using SEM (JEOL JSM 6510 La, Tokyo, Japan). The composite films were cut into 4 mm × 4 mm size, and the morphological images of the surface and the cross-section of the composite films were photographed at a magnification of 5000×.

#### 2.3.2. Fourier Transform Infrared (FTIR) Spectroscopy

The structure and function of group analysis of SA, SA/KC, and crosslinked SA/KC films were analysed using FTIR (Perkin-Elmer PC1600, Houston, TX, USA). FTIR spectra were recorded at a wavenumber of 4000–400 cm^−1^. The sample piece was ground with KBr salt at a ratio of 1:10 before analyzing [19]. Samples were put in FTIR cells, and the IR absorptions were recorded for functional group analysis.

#### 2.3.3. X-ray Diffraction (XRD) Analysis

For X-ray diffraction (XRD) analysis, the film samples were folded 20 times to increase the sample thickness [2]. Samples were analysed at 2θ of 10° to 90° with an angle step size 2θ = 0.02° in an XRD instrument [Shimadzu series 7000, Koriyama, Japan] using a Cu K˛ (30 kV/30 mA) source.

### 2.4. Mechanical Properties

The film sample mechanical properties analysis measures the Tensile strength (MPa) and deformation of the film using the tension method. Tensile strength was measured at 27 °C with a Testometric Machine M350-10CT (Testometric Co., Ltd., Rochdale, Lancs, UK) according to American Society for Testing and Materials (ASTM) ID: D882-12. All the tested were tested by the same size (5 cm × 10 cm) and was equilibrated at 28 °C and 87% RH in desiccators containing silica crystals to extract moisture content-saturated solutions for 48 hours prior to testing [4].

### 2.5. Physical Properties

The physical properties of the sample were observed by measuring the water solubility. The cast film was cut into a similar size (5 × 5 cm) and then stirred with constant speed in 50 mL of distillate water for 24 h at 25 °C. The undissolved film was then filtered and dried at 110 °C. The dried undissolved film was weighed until a constant weight was achieved [20]. The water solubility of the tested edible film was calculated by using Equation (1).

(1) $$W_{s}=\frac{W_{0}-W_{f}}{W_{0}}\times 100\%$$

where *W_S_* is water solubility (%), *W*_0_ is the initial weight of the sample (g), *W_f_* is the constant weight of the undissolved sample (g).

### 2.6. Antioxidant Analysis

The antioxidant activity of the films was evaluated through DPPH radical scavenging capability [3]. The film sample was filled with the DPPH containing solution and closed tightly. The mixture was incubated for 1 h in the low light chamber at room temperature. The absorbance of the DPPH solution was measured using an ultraviolet–visible (UV-Vis) spectrophotometer at a wavelength of 517 nm. The antioxidant activity was calculated through DPPH colour degradation, as expressed by its absorbance using Equation (2).

(2) $$Scavenging\left(\%\right)=\frac{A_{\left(blank\right)}-A_{\left(sample\right)}}{A_{\left(blank\right)}}\times 100\%$$

where *A_(blank)_* is the absorbance of the DPPH solution without sample and *A_(sample)_* is the absorbance of the DPPH solution with the soaked sample.

### 2.7. Antimicrobial Properties Evaluation

The antimicrobial activity was evaluated through the inhibition zone of the prepared edible films on the microbial growth media. To achieve this purpose, 10 µL of *Escherichia coli* (Persian type culture collection (PTCC) No. 1330) from the strain culture stock (10^6^ CFU/mL) was inoculated on the sterilized agar media growth (1.5 g of agar media in 100 mL distilled water). Then the tested film piece (15 mm in diameter) was placed in the center of the agar growth medium. The inoculum was incubated for 3 days at 30 °C. After the incubation, the average radius of inhibition coverage (*n*) was measured using a caliper, and the inhibition zone area was then calculated using Equation (3) [6].

(3) $$inhibitionzone\left({\mathrm{mm}}^{2}\right)=\frac{22}{7}\times n^{2}$$

### 2.8. Statistical Analysis

The statistical analysis of the experimental data was performed using analysis of variance (ANOVA) with the assistance of STATISTICA^TM^ software version 12 (Statsoft Inc., Hamburg, Germany). Significant differences among the variables were determined using Duncan’s multiple range test at a 95% confidence level.

## 3. Results and Discussion

### 3.1. Morphology Surface Observation

**Figure 2:**
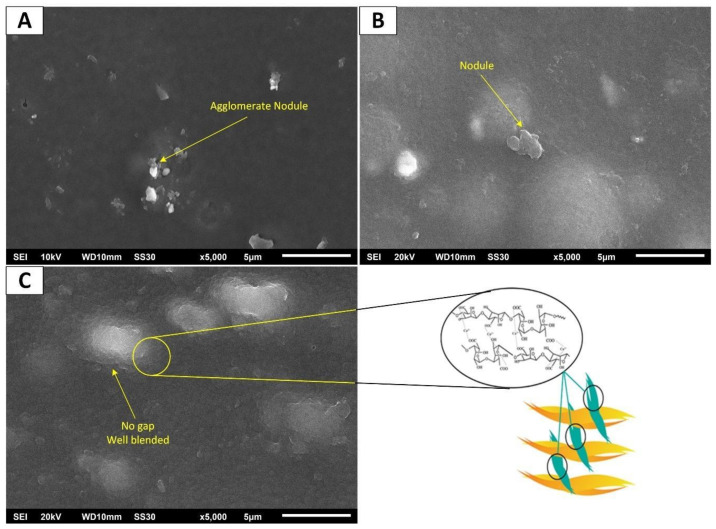
Surface scanning electron microscope (SEM) image of (**A**) pristine sodium alginate (SA) film; (**B**) sodium alginate/k-carrageenan (SA/KC) film; (**C**) Ca crosslinked SA/KC film.

Film surface morphology behavior was evaluated using SEM. The pristine SA, SA/KC, and crosslinked SA/KC film surface micrographs are presented in Figure 2. The SA film surface morphology, as shown in Figure 2A, exhibits agglomerated nodules of insoluble SA particles. This phenomenon could be due to the hydrophilic nature of SA, which possesses low solubility in water. The SA/KC film surface micrograph, as depicted in Figure 2B, is smoother and has fewer nodules. The agglomerate nodule on the film surface is undesired because it potentially initiates the film crack, thereby decreasing the physical properties. However, the SA/KC shows better surface morphology than that of pristine SA film. This can be attributed to the development of certain molecular aggregations of KC at the film surface with a further increase of KC. KC is a hydrophilic polysaccharide that has an excellent property to be soluble in water, while SA is a slightly hydrophobic polysaccharide due to the high concentration of the carboxyl group, thereby being less soluble in water than carrageenan.

On the other hand, it is also possible that the presence of KC strongly influenced the film’s surface features. It is concluded that the KC blending into SA could enhance the solubility degree of SA polymer to achieve a homogenous solution. Yang et al. [21] also reported the similar result for the incorporation of gelatin into SA film solution that showed smoother surface area without defected surface.

The surface morphology of Ca crosslinked SA/KC Film at CaCl_2_ concentration of 2.0 *w*/*v*-% was presented in Figure 2C. The surface morphology cross-linked film is denser, and no significant nodule is observed. It might be due to the Ca^2+^ crosslinker that could form an interlayer film between the alginate chains through carbonyl bonding with Ca^2+^. The interlayer formation reduces the free volume of the film matrix and creates the two-dimensional film with the interaction of each layer of the film and makes the film denser [19,21,22]. A similar result was also obtained by Wu et al. [23], where a smoother surface and more compact structure of crosslinked agent CaCl_2_ films were observed than non-crosslinked films. 

### 3.2. Fourier Transform Infrared (FTIR) Spectra Analysis

**Figure 3:**
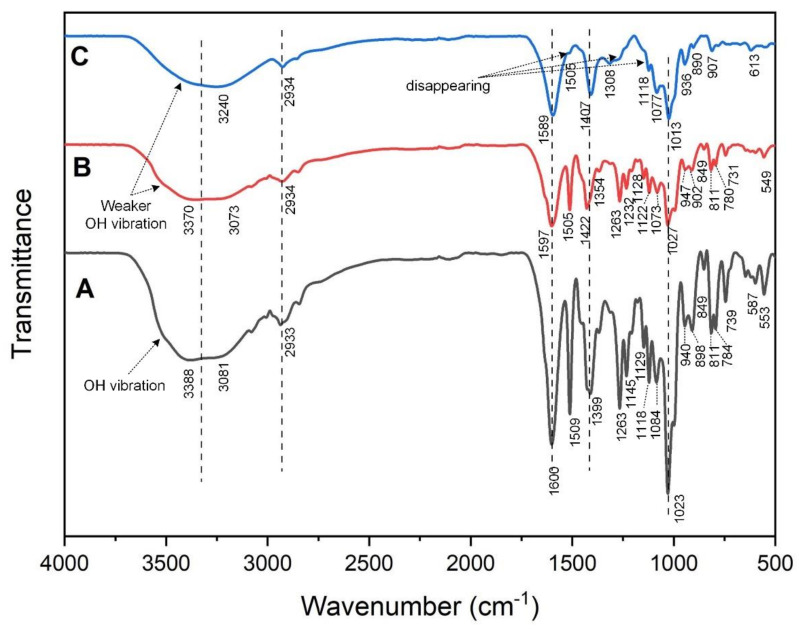
Fourier transform infrared (FTIR) spectroscopy observation result: (**A**) control film (SA); (**B**) SA/KC film; (**C**) crosslinked SA/KC film.

FTIR analysis of films is attempted to characterize KC incorporation and Ca crosslinking in the SA-based film matrix by distinguishing the bands of infrared (IR) absorption shifts related to SA/KC interactions. Figure 3A shows the FTIR spectra of the control film (pristine SA). A wide broad, strong peak at 3388 cm^−1^ is observed, which indicates the vibration of the hydroxyl functional group in the SA molecule. This typical peak was also found in Figure 3B,C at 3370 cm^−1^; however, with lower absorbance. Weaker hydroxyl vibration of SA/KC films may be influenced by the lower total volume occupation of alginate since alginate has more hydroxyl groups than carrageenan. The crosslinked SA/KC exhibits the lowest OH vibration and peak shifting at 3240 cm^−1^; this phenomenon could be due to the substitution of protons in carbonyl and sulfonyl groups with calcium ions during the crosslinking process, thus significantly decreasing the amount of hydroxyl in the polymer chain. This result is in agreement with the previous report by Giz et al. [17]. The peak at 2934 cm^−1^ was found in all film types belonging to the saturated C–H stretching.

**Figure 4:**
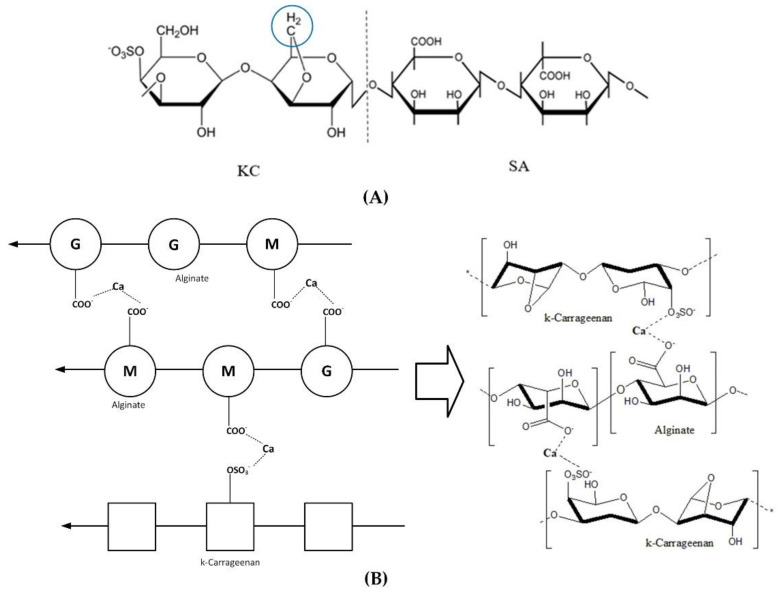
Scheme of (**A**) SA/KC film reaction, and (**B**) SA/KC interaction through Ca^2+^ crosslinking.

The confirmation of Ca^2+^ crosslinking of SA-SA and SA-KC is evaluated by indicating the peak intensity alteration, shifting, and disappearance between SA/KC film spectrum (Figure 3B) and the crosslinked SA/KC spectra (Figure 3C). Three peaks disappear in the FTIR spectra of crosslinked SA/KC (Figure 3C) at 1505, 1263, and 1128 cm^−1^, which are considered as sulfonyl stretching, and secondary hydroxyl stretching. These groups contributed to the crosslinking process with Ca^2+^ ions. Crosslinked SA/CA film also shows a peak at 1121 cm^−1^ that belongs to C–O–C symmetry, stretching of SA polysaccharide and polymerization between SA and KC to create a longer chain, as shown in Figure 4A. The IR absorption at 1597 and 1422 cm^−1^ are important peaks to confirm the crosslinking process of the film. The lower peak at 1604 cm^−1^ to 1595 cm^−1^ was attributed to the asymmetric and asymmetric stretching vibration of the SA carboxyl group (–COO–) and shifting peak from 1422 cm^−1^ to 1407 cm^−1^ due to the cationic substitution in the carboxyl group of SA. The peak intensity decreases at 1263, and 1232 cm^−1^ are related to the crosslinking of the divalent ion Ca^2+^, which creates a stronger bond with anionic COO–. Figure 3C also shows the intensity at 907 cm^−1^, which is attributed to the crosslinking process of the film, which substituted the C–H bond at 1,4-disubstituted or 1,2,3,4-tetrasubstituted, creating the denser film. The peak alteration and shifting, as shown in crosslinked SA/KC FTIR spectra, were attributed to the interaction of Ca^2+^ ion with the anionic region of SA and KC [16] that form a complex network as illustrated in Figure 4b. A weak peak observed at 1505 cm^−1^ in the crosslinked SA/KC spectra corresponds to the C=C stretching of the aromatic ring in the incorporated CEO [14].

### 3.3. X-ray Diffraction (XRD) Pattern Analysis

**Figure 5:**
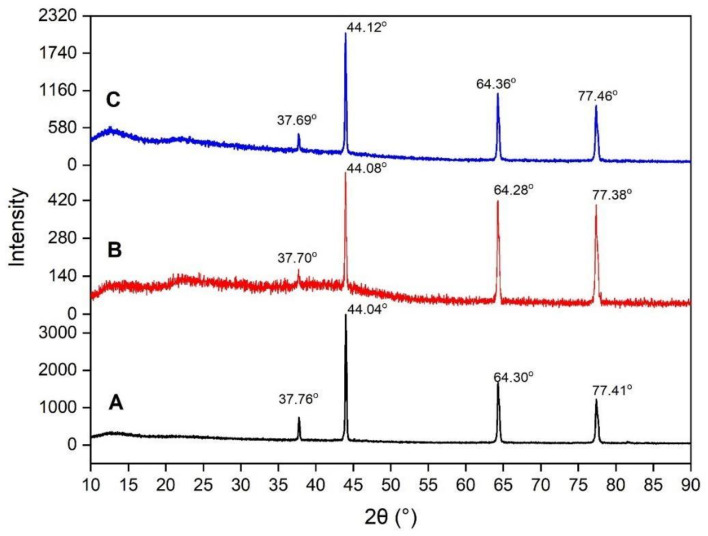
The X-ray diffraction (XRD) pattern of (**A**) control film (SA); (**B**) SA/KC film; (**C**) Crosslinked SA/KC film.

**Figure 6:**
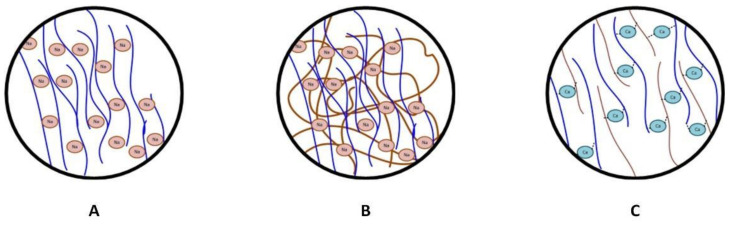
Simplified illustration of SA/KC crosslinking mechanism using Ca: (**A**) sodium alginate structure, (**B**) sodium alginate/ κ -carrageenan mixture, (**C**) Ca crosslinked alginate/κ-carrageenan structure.

X-ray diffraction pattern analysis was performed to evaluate the crystal properties improvement of the crosslinked SA/KC film. Figure 5 presents the XRD pattern for pristine SA, SA/KC, and crosslinked SA/KC at 2θ from 10° to 90°. The XRD spectra of pristine SA film have shown a typical characteristic of polycrystalline materials. The crystalline peaks at 2θ of 37.69°, 44.12°, 64.36°, and 77.46° are originally from the alginate crystal structure. A similar result was also obtained by Faried and co-workers [24] and they also found that four distinct diffraction peaks at 38.26°, 44.47°, 64.71°, and 77.74° could be assigned as sodium alginate typical peaks. However, the KC loading in SA film decreases the film crystallinity and the crosslinking of Ca^2+^ seems to have repaired it. Based on the peak integration, the crystallinity index (CI) of pristine SA, SA/KC, and crosslinked SA/KC films are 89.91%, 28.70%, and 43.57%, respectively. The XRD pattern of SA/KC film as presented in Figure 5B shows the decrease of crystalline peak at 37.70° indicating the film is more amorphous than that of pristine SA. This could be due to the amorphous biopolymer properties of KC, thereby the addition of KC could decrease the crystallinity degree of the film. For SA/KC film with the presence of CaCl_2_ crosslinker as shown in Figure 5C, the recorded XRD patterns indicate the interaction of Ca^2+^ caused the improvement of CI by increasing the CI of the film from 28.70% to 43.57%. The intermolecular interactions in SA/KC in the presence of Ca^2+^ crosslinker agents were further strengthened due to interlayer film formation. The illustration of polymer structure improvement due to the interaction of Ca^2+^ with SA and KC in the film matrix is presented in Figure 6. The ionic bonding of Ca^2+^ with carboxyl and sulfonyl formed a crosslink network that increased the regularity degree of polymer chains in the film, and thus improving the CI of the film. The enhancement of CI of the film could contribute in improving the physical features and mechanical properties.

### 3.4. Mechanical Properties of Prepared Films

Tensile strength and elongation at break are useful in predicting the edible film’s ability to maintain its integrity when used as food packaging. This research was to observe the effect of KC incorporation into the SA film. Table 1 presents the tensile strength of the control film (SA), and SA/KC films were 23.02 MPa and 20.96 MPa, respectively. The elongation at break percentage of control film was 8% and SA/KC (9/1) film was 86%. Furthermore, Table 1 also shows that tensile strength of the ratio of SA/KC powder decreased from 20.96 MPa to 16.77 MPa along with the increasing KC loading, however, the elongation at break significantly increased from 26% to 86%. The result shows that the addition of KC into the SA film solution could decrease tensile strength and increase the elongation at break of film. It could be explained that SA contains divalent ions of (Na) that could express crystalline form due to the mineral compound/divalent ions contained in the film and this divalent ion could create an ionic bond which is stronger than the covalent bond and contains a large amount of carboxyl groups. These results are supported with XRD characterization where the CI of the film was significantly decreased by the incorporation of KC. The CI of the film is strongly correlated with the mechanical properties of the film; the crystalline phase tends to increase the stiffness and tensile strength, while the amorphous phase is more effective in absorbing impact energy (higher EB percentage) [25]. This result was also supported by Paşcalău et al. [26] that reported that alginate has a higher tensile strength than kappa-carrageenan, however, Paula et al. [27] reported the contradictory result that a higher proportion of KC in the film mixture possessed higher tensile strength and Young’s modulus. Yu, et al. [28] reported that KC has high gel properties that could plasticize the film. Increasing the concentration of carrageenan in the SA film, which reduces tensile strength and improves the elongation at break of the film. Yang et al. [21] reported that SA/gelatine weight ratio varied from 10:0 to 5:5, the tensile strength decreased from 42.05 MPa to 32.34 MPa.

The addition of CEO into SA/KC film could affect both tensile strength and elongation at break of the SA-based film shown in Table 1. The film was prepared with SA/KC with a ratio of 9/1, and then the concentration of CEO loading varied from 1.5 to 3.0% *v*/*v*. The tensile strength measurement shows that tensile strength decreases from 28.46 to 20.96 MPa and the elongation at break increases from 58% to 70%. The possible answer is that the SA/KC film has low affinity to interact with hydrophobic compounds such as essential oils due to the strong electrolyte properties of SA/KC molecules. Therefore, the presence of the CEO in the SA/KC matrix hinders the polymer–polymer intermolecular attraction. Even if the CEO loading decreases the mechanical properties of the film, the tensile strength and elongation break values are still in the acceptable threshold. Recent study shows that essential oil has the ability to improve the plasticization property of the film [14]. Ahmed et al. reported that the presence of EO reduced the tensile strength to 10.93 MPa and a significant improvement in the elongation at break to 204% of the film [14]. The result of this research shows the incorporated KC and CEO into the SA film formulation could decrease the mechanical properties. There were several research reports that the addition of crosslink agents such as CaCl_2_, AlCl_3_, BaCl_2_, etc. could improve the mechanical properties of films [29].

In this study, the improvement of the mechanical properties of SA/KC films was carried out with CaCl_2_ crosslinking agent. The film was prepared with a SA/KC ratio of (9/1) and CEO concentration was 3% *v*/*v*, while the concentration of CaCl_2_ varied from 0% to 2% *w*/*v*. The tensile strength and elongation at break percentages of crosslinked SA/KC films with various CaCl_2_ concentrations are presented in Table 1. The increase of CaCl_2_ concentration could enhance the tensile strength from 20.96 to 26.21 MPa and reduce elongation at break from 70% to 20%. Crosslink agents containing Ca^2+^ could interact with COO–molecules of the SA group which occurred within the interlayer of the film which makes the film denser because each layer of the film interacts one another with the presence of Ca^2+^ and created an ionic bond between the layers [19]. Costa et al. [30] reported that the reaction during the crosslinking process created the structure that reduced the free volume area of the film and produced a stronger film. They also explained that the degree of crosslinking depends on the ability of the crosslinking ion to diffuse through the film. As shown in XRD interpretation, the interaction of Ca^2+^ with anionic parts of the SA and KC significantly improves the CI of the matrix, where CI is strongly correlated with the mechanical properties of the film.

It can be described that CaCl_2_ alkaline suspension treatment promotes intermolecular interaction and bonding in SA/KC in the presence of crosslinker agents. The formation of an interlayer crosslinking network structure by Ca^2+^ crosslink agents was created by the ionic bond between Ca^2+^ and COO^-^ as supported by FTIR interpretation in the previous section. The result indicates that the crosslinker agent CaCl_2_ in the film blends created a close interaction among carboxyl of SA, sulfonyl of KC, and calcium ions, thereby increasing the tensile strength and reducing the elongation at break of the film. Li. et al. [16] reported similar results that when Ca^2+^ content was 1.5% w/v, tensile strength could reach 166.90 MPa while the elongation at break had a converse trend.

### 3.5. Physical Properties

The physical properties of the prepared edible film were evaluated by using film thickness and water solubility. The film thickness plays an important role in determining the mechanical properties in terms of tensile strength of the film. The effects of incorporating CEO and CaCl_2_ crosslinker agents on the physical properties of SA/KC films are presented in Table 1. The result shows the thicknesses of the SA/KC (9/1) film and the control film (SA) are 0.15 and 0.37 mm, respectively. At a higher ratio of KC in the SA matrix (9/1 to 6/4), the thickness of the film decreases from 0.15 mm to 0.08 mm. Table 1 also shows the SA/KC film (9/1) with the presence of CEO (1.5% to 3.0% *v*/*v*) increasing the film thickness from 0.09 mm to 0.15 mm with respect to the CEO concentration. The SA/KC Film (9/1) with 3% *v*/*v* CEO and varied CaCl_2_ concentration (0% to 2% *w*/*v*) of crosslinker solution significantly increased the film thickness from 0.15 to 1.1 mm along with the increase of CaCl_2_ concentration.

Physical properties of the film were also determined using water solubility. It was beneficial when the integrity of the product and water resistance film were desired. Table 1 shows that the water solubility value of SA/KC increased from 47.94% to 59.04% for the SA/KC ratio of 10/0 to 6/5, respectively. KC is a natural polysaccharide containing a linear chain of sulfonates (galactose and 3,6 anhydro galactose groups). The content of sulfate groups causes carrageenan to be hydrophilic, which is water soluble, while SA is a hydrophobic compound that is not easily dissolved in water. Higher ratio of SA/KC film caused the improvement of water solubility of SA/KC film due to the higher hydrophilic properties of the SA/KC film. On the other hand, SA is less soluble in water properties, which could increase water solubility by reducing the concentration of SA. Mohammadi et al. also reported that the presence of SA decreased the water solubility of SA/gelatin film [31].

Table 1 also shows the effect of CEO on the water solubility properties of the SA/KC film. The results of this study indicate that increasing the concentration of CEO will slightly reduce the value of water solubility edible film SA/KC. The water solubility of SA/KC film decreased from 59.04% to 52.90% with the increase in CEO content from 1.5% to 3% *v*/*v*. This is due to the increased CEO content caused by the non-polar solution of the CEO that has hydrophobic properties; this can contribute to reducing the molecular interactions between the film particles caused by the insolubility of the SA/KC solution and the non-polar solutions.

The presence of CEO also could reduce the interaction of the polysaccharide with the hydroxyl group of the film. In the other word, the presence of the CEO increases the hydrophobicity of film. More hydrophobic behavior of film reduces the solubility in water [3,5,6]. Hosseini et al. [29] reported that the presence of the CEO on the chitosan-based film decreased the water solubility from 27.74% to 21.52%. A similar pattern was also reported by Shojaee-Aliabadi et al. [4] that the water solubility of carrageenan-based film was reduced during the addition of essential oil (1% to 3% *v*/*v*) from 20.85% to 16.09%. Mahcene et al. [32] revealed that the water content in the films provided an indication of the film hydrophilicity, where the higher water solubility value indicates higher hydrophilic property of the film.

The variation of the concentration of CaCl_2_ in the crosslinker solution exhibits a noticeable effect on the water solubility value as presented in Table 1. The increase of CaCl_2_ concentration in the crosslinker solution was observed to be lowering the water solubility degree of the film from 51.49% to 28.49%. Increasing the number of crosslinking points in the polymeric chain will allow the water to be maintained at a higher crosslinking density and the copolymer to become less soluble. The crosslinkers can significantly reduce water solubility due to the utilization of the electrolyte region of SA and KC as the crosslinking network as revealed by FTIR interpretation in the previous discussion. It has also been reported that crosslinking of alginate with CaCl_2_, can cause an interaction between Ca^2+^ and anionic alginate to build ionic bonds and the film interlayer, which reduces the hydroxyl affinity [16,19].

### 3.6. Antioxidant and Antimicrobial Activity

**Figure 7:**
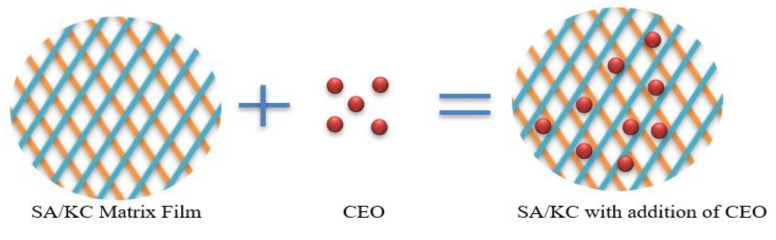
The scheme of incorporated eugenol group contained in clove essential oil (CEO) in the film matrix.

The antioxidant and antimicrobial activity of the film is provided by the incorporation of bioactives containing CEO. Based on the FTIR interpretation analysis, there was no chemical reaction between the CEO with SA/KC matrix film due to its nonpolar nature. The CEO molecule is only trapped among the polymer matrix as illustrated in Figure 7. The CEO contains bioactive components such as eugenol and caryophyllene which have antioxidant, antifungal, and antimicrobial behavior. The incorporation of bioactives containing essential oils into an alginate-based films is expected to improve the antioxidant and antimicrobial properties.

The eugenol trapped in the SA/KC film matrix would work as antioxidant and antimicrobial activity, because SA/KC has good properties for rapidly releasing the contents trapped in the SA/KC film matrix. DPPH radical scavenging and bacteria growth inhibition zones are performed to evaluate the performance of antioxidant and antimicrobial activity of the prepared film.

#### 3.6.1. DPPH Radical Scavenging Assay

CEO with eugenol as the main bioactive compound serves as antioxidants by interrupting chain oxidation reactions and contributing a hydrogen atom as a free radical acceptor, or by chelating metals. To assess the antioxidant activity of CEO incorporated SA/KC film, DPPH scavenging is effective due to its fast, convenience, simplicity, and accuracy [20]. This method is commonly used to determine the free radical scavenging ability of many phenolic bioactive compounds.

**Figure 8:**
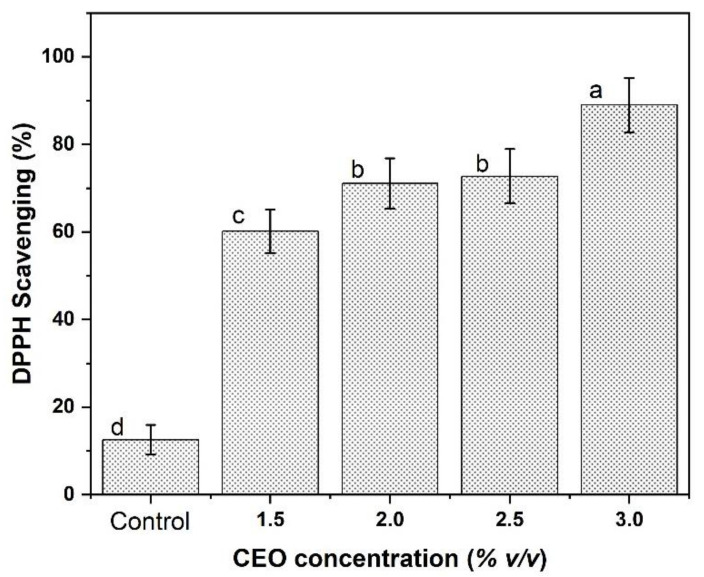
The effect of addition eugenol group contained in CEO incorporated into SA/KC film matrix against DPPH activity. ^a,b,c,d^ The reported data are the average and standard deviations where values in each bar with different letters are significantly different (*p* < 0.05).

Figure 8 shows the addition of CEO at various concentrations (1.5%; 2.0%; 2.5%; 3.0% *v*/*v*) into the SA base. The result confirmed the effectiveness of 3% of CEO as an antioxidant could reach 90.32% of DPPH scavenging. According to a study reported by Ahmed et al. [14], the antioxidant activity of essential oils is caused by the presence of phenolic groups. This antioxidant property of the films could be explained due to phenolic compounds in CEO such as eugenol, gallic acid, etc. The hydroxyl groups attached to the aromatic ring of phenolic compounds act as electrophilic that capture lone-paired electrons from free radicals and are trapped in the electron cloud of the aromatic ring. While a higher concentration of CEO could also improve the antioxidant activity, according to this work, the DPPH scavenging activity increased from 60.94% to 90.32% with 3% *v*/*v* CEO loading. These findings suggested a higher antioxidant potential due to the reaction of phenolic compounds with radicals.

Previous researchers also reported the effectiveness of essential oil incorporation into the alginate-based matrix film for improving the antioxidant activity. Salarbashi et al. [33], reported that the DPPH activity increased from 42.40% to 80.60% using *Zataria multiflora* essential oil (1% to 3% *v*/*v*). Ballester-Costa et al. [34] also reported that the DPPH activity was increased during the presence of various thymus essential oils, which improved to 55% of DPPH activity. Dou et al. [19] also confirmed the effectiveness of phenolic compounds contained in tea extract that are effective to improve the antioxidant activity. However, it is not only the major constituents of essential oil that are responsible for this antioxidant activity, there may also be other minority compounds that can interact in a synergistic or antagonistic way to create an effective system-free radical.

#### 3.6.2. Inhibition Zone

The inhibition zone indicates the ability of essential oils to provide antimicrobial properties by inhibiting microbial growth in a specific area. Phenolic compounds in the CEO migrate from the film matrix into the microbial growth medium as an antimicrobial agent. The results of the antimicrobial activity of the film at various concentrations of CEO loading are shown in Table 2. The inhibition area increases with respect to the increase of CEO loading concentration. This study has shown that with a higher level of CEO concentration in the film, the antimicrobial activity increased from 28.28 to 113.14 mm^2^ of *E. coli* bacterial growth. Improving antimicrobial activity could be caused by the content of eugenol groups that contain in the CEO. Eugenol contained in CEO is one of the phenolic groups that has been modified, which has the properties as an antimicrobial agent that could be considered as the source of lower and reducing microbial activity of the SA/KC films. It is assumed that eugenol is the principal inhibitory component of the essential oil in the clove. Sublethal concentrations of eugenol and cinnamaldehyde by *Bacillus cereus* have been found to inhibit amylase production and proteases. The weakening of the cell wall and a high degree of cell lysis were also noticed [29]. It has been considered that the CEO is affected by the microbial activity of the SA/KC films. It was shown that higher levels of CEO can lead to decreased micro activity. Table 2 shows the inhibition zone of the film during the presence of the CEO against *E. coli* bacteria.

The scheme of improving the inhibitor zone during the addition of essential oil, in this case CEO, could be attributed to the ability of SA/KC film, which has a good rapid release property where eugenol contained in CEO is trapped in the SA/KC film matrix once the bacteria penetrates the film solution; the film is influenced by the rapid release of the volatiles compound contained in CEO where eugenol not only as the main compound of the CEO but also the volatile content that contains in CEO. The rapid release of eugenol content from the SA/KC film solution could inhibit the growth of *E. coli* bacteria. A higher amount of eugenol compounds in the film also spread the inhibition zone of the film larger during the increase in CEO concentration from 1.5% to 3%, where the inhibitor diameter increases from 3 mm to 6 mm. Another study also reported that the antimicrobial activity of nanoparticles and essential oils against *E. coli* had been established earlier [35,36]. The hydrophobic compounds from the essential oil and zinc cations can penetrate through the bacterial cell membrane to rupture the cell structure [32].

## 4. Conclusions

Modified SA/KC film has been successfully fabricated in this study. The increase of the KC ratio in the SA-based matrix significantly decreased the mechanical properties of tensile strength. However, it increased the power absorption or elongation at break (flexibility). The presence of KC caused the decrease in the crystallinity index indicated by XRD and FTIR results. The addition of KC could improve the morphology surface of the film as shown in SEM images. The addition of the CEO into the matrix network of the SA/KC film reduces the mechanical properties but shows an improvement in film flexibility. Furthermore, the crosslinking process was carried out to improve the physical and mechanical properties of the film. The addition of CaCl_2_ formed an interlayer film by the formation of O–Ca–O groups. It also increased the crystalline index thereby improving the physical and mechanical properties of the SA/KC film. The addition of CEO into the matrix film also showed effectiveness in increasing the antioxidant and antimicrobial properties of SA/KC films.

## Figures and Tables

**Table 1:** Mechanical and physical properties of the SA/KC films.

Variable	Conc.	Thickness (mm)	Tensile Strength (MPa)	Water Solubility (%)	Elongation at Break (%)
SA/KC	10/0	0.37 ± 0.018 b	23.02 ± 1.15 c	47.94 ± 3.43 d	8 ± 0.39 j
	9/1	0.15 ± 0.012 c	20.96 ± 1.09 d	52.90 ± 1.47 c	26 ± 0.21 g
	8/2	0.11 ± 0.052 d	20.65 ± 0.78 d	54.98 ± 0.61 b	46 ± 0.10 f
	7/3	0.10 ± 0.062 d	17.93 ± 1.93 e	57.00 ± 2.62 a	70 ± 0.23 b
	6/4	0.08 ± 0.008 e	16.77 ± 1.09 e	59.04 ± 4.67 a	86 ± 0.39 a
CEO (*v*/*v*)	1.5%	0.09 ± 0.017 d	28.46 ± 1.29 a	59.04 ± 3.06 a	58 ± 0.60 e
	2.0%	0.11 ± 0.002 d	25.97 ± 1.80 b	57.00 ± 1.02 a	60 ± 0.40 d
	2.5%	0.13 ± 0.022 c	24.32 ± 0.15 c	54.98 ± 0.99 b	68 ± 0.40 c
	3.0%	0.15 ± 0.012 c	20.96 ± 1.09 d	52.90 ± 1.47 c	70 ± 0.60 b
CaCl_2_ (b/v)	0%	0.15 ± 0.017 c	16.77 ± 1.69 e	51.49 ± 1.80 c	86 ± 0.44 a
	1%	1.00 ± 0.030 a	24.41 ± 0.94 c	39.11 ± 0.57 e	20 ± 0.22 i
	2%	1.10 ± 0.013 a	26.21 ± 0.74 b	28.45 ± 1.23 f	22 ± 0.20 h

The reported data are the average and standard deviations where values in each column with different letters are significantly different (*p* < 0.05).

**Table 2:** Inhibition zone of CEO-incorporated SA/KC film.

CEO Concentration (%v)	Radius (mm)	Inhibition Area (mm^2^)
0.00 (control)	0	0.00 ± 0.00 ^e^
1.50	3	28.28 ± 3.29 ^d^
2.00	4	50.28 ± 1.29 ^c^
2.50	5	79.57 ± 2.00 ^b^
3.00	6	113.14 ± 5.57 ^a^

^a–e^ The reported data are the average and standard deviations where values in each column with different letters are significantly different (*p* < 0.05).
